# Whole genome association study identifies regions of the bovine genome and biological pathways involved in carcass trait performance in Holstein-Friesian cattle

**DOI:** 10.1186/1471-2164-15-837

**Published:** 2014-10-01

**Authors:** Anthony G Doran, Donagh P Berry, Christopher J Creevey

**Affiliations:** Teagasc Animal and Bioscience Research Department, Animal & Grassland Research and Innovation Centre, Teagasc, Grange, Dunsany, Co, Meath, Ireland; Molecular Evolution and Bioinformatics Unit, Biology Department, NUI Maynooth, Maynooth, Co, Kildare, Ireland; Teagasc Animal and Bioscience Research Department, Animal & Grassland Research and Innovation Centre, Teagasc, Moorepark, Fermoy, Co, Cork, Ireland; Institute of Biological, Environmental and Rural Sciences, Aberystwyth University, Aberystwyth, SY23 3FG UK

**Keywords:** Genome-wide association, Single nucleotide polymorphism, Holstein-Friesian, Carcass, Biological pathways

## Abstract

**Background:**

Four traits related to carcass performance have been identified as economically important in beef production: carcass weight, carcass fat, carcass conformation of progeny and cull cow carcass weight. Although Holstein-Friesian cattle are primarily utilized for milk production, they are also an important source of meat for beef production and export. Because of this, there is great interest in understanding the underlying genomic structure influencing these traits. Several genome-wide association studies have identified regions of the bovine genome associated with growth or carcass traits, however, little is known about the mechanisms or underlying biological pathways involved. This study aims to detect regions of the bovine genome associated with carcass performance traits (employing a panel of 54,001 SNPs) using measures of genetic merit (as predicted transmitting abilities) for 5,705 Irish Holstein-Friesian animals. Candidate genes and biological pathways were then identified for each trait under investigation.

**Results:**

Following adjustment for false discovery (q-value < 0.05), 479 quantitative trait loci (QTL) were associated with at least one of the four carcass traits using a single SNP regression approach. Using a Bayesian approach, 46 QTL were associated (posterior probability > 0.5) with at least one of the four traits. In total, 557 unique bovine genes, which mapped to 426 human orthologs, were within 500kbs of QTL found associated with a trait using the Bayesian approach. Using this information, 24 significantly over-represented pathways were identified across all traits. The most significantly over-represented biological pathway was the peroxisome proliferator-activated receptor (PPAR) signaling pathway.

**Conclusions:**

A large number of genomic regions putatively associated with bovine carcass traits were detected using two different statistical approaches. Notably, several significant associations were detected in close proximity to genes with a known role in animal growth such as glucagon and leptin. Several biological pathways, including PPAR signaling, were shown to be involved in various aspects of bovine carcass performance. These core genes and biological processes may form the foundation for further investigation to identify causative mutations involved in each trait. Results reported here support previous findings suggesting conservation of key biological processes involved in growth and metabolism.

**Electronic supplementary material:**

The online version of this article (doi:10.1186/1471-2164-15-837) contains supplementary material, which is available to authorized users.

## Background

Animal growth is an economically important trait for livestock raised for meat production. Carcass traits, related to animal growth, are critical to the biological and economical efficiency of cattle production and, as such, there is great interest in understanding the underlying genomic architecture influencing these traits. Quantitative trait loci (QTL) associated with a particular trait can be used to predict disease risk or genetic merit of an animal [1, 2]. This information may also be used to investigate the molecular mechanisms and biological pathways involved in phenotypic variation between animals. Investigating complex traits in domestic animals may also provide insights into mechanisms underlying similar traits, such as growth and fat deposition, in humans.

Holstein-Friesian cattle are a popular breed of cow primarily used for their ability to produce large quantities of milk. However, Holstein-Friesian cattle are also an important source of meat for beef production. Several studies in cattle have identified associations between carcass traits and regions of the bovine genome. Carcass trait QTL have been reported most often on chromosomes 2, 3, 6, 14, 20 and 29 [3–7]. However, most studies reporting carcass QTL have been performed using beef breeds such as Aberdeen Angus [3]. There have been no studies to date that have investigated the association of SNP genotypes with carcass performance utilizing measures of genetic merit estimated in dairy breeds. Although many studies have reported carcass QTL in regions containing genes with a known role in animal growth (such as the myostatin gene on bovine chromosome 2 [8, 9]), little is known about the mechanisms or underlying biological pathways involved in growth or carcass traits. Moreover, many of the reported QTL have been identified using raw phenotypic data and how the phenotypic data reflects the underlying genetic merit of the animal is a function of the heritability. By using estimates of individual animal genetic merit, generated from the accumulation of phenotypic information on relatives, the accuracy of the phenotype can be considerably greater and thus the statistical power of the association study is greater for the same number of genotyped individuals.

The objective of this study was to identify regions of the bovine genome associated with carcass performance traits using two statistical approaches: a single marker regression and multi-locus Bayesian approach. Regions detected as associated with a trait were then further investigated to identify the potential causal pathways and biological processes underlying each trait.

## Methods

### Ethics statement

Semen samples for genotyping were collected by the Irish Cattle Breeding Federation [10] and partner artificial insemination organizations. All animal procedures were carried out according to the provisions of the Irish Cruelty to Animals Act (licenses issued by the Department of Health and Children).

### Genotypic data

Genotypes of 54,001 biallelic single nucleotide polymorphism (SNP) markers from 5,706 Holstein-Friesian sires were available for use in this study. All genotyping was carried out using the Illumina Bovine SNP50 version 1 Beadchip (Illumina Inc., San Diego, CA; [11]). SNP positions were based on the Btau 4.0 assembly of the bovine genome. All SNPs on the X-chromosome or with an unknown position in the genome were removed from the dataset. Quality filtering was then undertaken to remove SNPs with inconsistent Mendelian inheritance patterns from sire to progeny. SNPs that had a minor allele frequency of less than 5% were also discarded. If a SNP had greater than 5% of calls missing, it was excluded from further analysis. Also, SNPs that failed to distinctly cluster into homozygous and heterozygous calls were removed. A total of 42,477 SNPs remained for analysis after quality filtering.

### Phenotypic data

Phenotypes for four economically important carcass traits were used in this study; carcass weight, carcass fat, carcass conformation of progeny and cull cow carcass weight. Carcass weight refers to the cold weight (measured in kgs) of the carcass taken within 2 hours of slaughter after being bled and eviscerated, and after removal of skin, external genitalia, the limbs at the carpus and tarsus, head, tail, kidneys and kidney fats and the udder. Progeny carcass weight is the carcass weight of a sire’s offspring/progeny measured on males from 300–1200 days and females from 300–875 days of age (females which have not produced a calf). Carcass fat and conformation phenotypes have been assessed since the year 2005 by video image analysis of the outside of the carcass [12] on a 15-point scale. Progeny carcass fat is the quantity of subcutaneous fat on the carcass of the slaughtered animal varying from 1 (leanest) to 15 (fattest). Progeny carcass conformation is the thickness of muscle on the carcass of the slaughtered animal scored on a scale of 1 (poor conformation) to 15 (excellent conformation). Cull cow carcass weight refers to the carcass weight of a dairy or beef cow slaughtered for meat at the end of her productive life. Cows are aged between 875 and 4000 days of age. Phenotypes for each of these traits are published as predicted transmitting abilities (PTAs), which are sire genetic merit based not on the sires themselves but on the performance of their descendants across multiple generations. Each PTA is accompanied by a respective reliability, which is the confidence in the estimated PTA (scale between 0 – 99%). As more information is included in an animal’s genetic evaluation, the reliability of the evaluation will increase. As the reliability increases, the likelihood that the animal’s PTA will change in the future is reduced as more information is included. The Irish Cattle Breeding Federation calculated PTAs and their respective reliabilities were available for all animals used in this study. Genotypic and phenotypic data for all animals utilized in this study can be requested from the Irish Cattle Breeding Federation [10]. The Irish Cattle Breeding Federation database identifiers for all animals used in this study are contained in Additional file 1. These animals were representative of the Holstein-Friesian population in Ireland. Phenotypic edits were then applied to the animal data. An adjusted reliability was estimated for each animal by removing the parental contribution to reliability as described by Harris and Johnson [13]. To ensure accurate phenotypes, for each trait separately, animals with an adjusted reliability of <70% were discarded. Following removal of animals with a low adjusted reliability, 1,061 animals remained for further analysis. Summary statistics for each of the phenotypes (as de-regressed PTA [14]), following removal of animals with an adjusted reliability of <70%, are in Table 1.

**Table 1:** **Summary statistics for the phenotypic data**

Trait	N	Min	Max	Mean	σ	Rel _min_	Rel _max_	Rel _mean_	Rel _σ_
CWT	941	−26.38	13.88	−4.07	6.39	70.2	99	89.55	8.36
CFAT	768	−0.72	0.62	−0.11	0.23	70.2	99	88.21	8.98
CONF	936	−1.62	0.46	−0.67	0.31	70.2	99	89.97	8.16
CULL	763	−29.44	29.65	0.33	8.28	70.2	99	88.14	8.37

Summary statistics include the total number of phenotype records (N), minimum value, maximum value, mean and standard deviation (σ) for each trait. Phenotypes are expressed as de-regressed predicted transmitting abilities. The minimum (Rel_min_), maximum (Rel_max_), mean (Rel_mean_) and standard deviation (Rel_σ_) for the associated adjusted reliabilities is also included.

CWT = carcass weight; CFAT = carcass fat; CONF = carcass conformation; CULL = cull cow carcass weight.

### Statistical analyses

Two statistical approaches, a frequentist (single SNP regression) and Bayesian approach (BayesB), were used to estimate associations between SNPs and each trait separately.

### Single SNP regression

The single SNP regression (SSR) model included each SNP separately as a continuous variable (i.e., count of a given allele) in a linear animal mixed model using ASReml [15]. The individual animal was included as a random effect. Relationships between animals were accounted for using the additive genetic relationship matrix. Pedigree information consisted of 6,854 animals. The dependent variable was de-regressed PTA [14]. Marker effects and associated P-values for each SNP were obtained from the analysis. P-values were adjusted to correct for errors arising from multiple testing using a false discovery rate (FDR) approach (FDR < 0.05) described by Storey and Tibshirani [16]. This procedure was carried out using the q-value package in R. Resultant q-values <0.05 were defined as significant. Adjacent SNPs, based on genomic location, that had q-values <0.05 were considered to be part of the same QTL. Genomic co-ordinates, identifier information, and q-values for all SNPs in the analysis are contained in Additional file 2.

### Bayesian approach

The second statistical approach utilized the Bayesian mixture model “BayesB” as described by Meuwissen *et al.* [17]. Source code for the BayesB software was provided by the author (Donagh P. Berry). A local version of BayesB was compiled on an in-house Linux server allowing us to efficiently carry out many parallel analyses. The Bayesian approach allows the incorporation of prior knowledge about the distribution of SNPs effects. An inverse chi-squared distribution (v = 4.234, S = 0.0429) was included in the model as the prior distribution of the mean and genetic variation of each SNP included in the model.

A prior value was assigned to π which describes a prior probability of association (1 - π) for each SNP. As this prior probability is assigned to all SNPs in the analysis, it reflects, *a priori,* the proportion of SNPs assumed to be associated with a particular trait. Analyses were run with alternative prior probabilities assumed to be associated with a particular trait (1- π) ranging from 0.05 to 6.25 × 10^−5^ (specifications of (1- π) are included in Additional file 3).

Additional analyses were also performed using the proportion of non-significant (q ≥ 0.05) SNPs that were estimated from the SSR analysis (pSSR), and half and double this value, to determine π. This was then used to quantify a prior proportion of SNPs assumed to be associated with each trait (1 – π). A total of eleven analyses were run for each trait. Markov Chain Monte Carlo (MCMC) chains were used to sample every 500^th^ iteration from the posterior distribution of SNP effects. Total iterations for each analysis are contained in Additional file 3.

#### Convergence testing

Convergence of the model for each analysis was confirmed by three approaches: Firstly a visual inspection of summed absolute log-likelihood values. All sampled iterations before convergence were discarded as burn-in. The number of iterations discarded as burn-in for each analysis are contained in Additional file 4. From the remaining sampled iterations, posterior probabilities (PPs) of association were calculated. A PP is the number of sampled iterations after burn-in that a SNP had a non-zero effect divided by the total number of sampled iterations after burn-in. The PP is indicative of the probability that a SNP is associated with a phenotype. A PP of zero indicates a low probability of association whereas a PP of 1 indicates a high probability of association.

The second approach used to ensure that convergence was successfully achieved, was performed by quantifying and plotting the total number of SNPs that had a PP > 0.5 at each iteration. The resultant trace plot was visually inspected to determine if the MCMC chains had run sufficiently long enough to have confidence that all high PP QTL had been detected.

Thirdly, the estimated marker effects for each SNP were checked for convergence. The combined difference between the estimated SNP effect of those SNPs with a PP > 0.5 from the Bayesian approach and the SNP effect for the same set of SNPs as estimated using the SSR approach was calculated using a Euclidean distance. Visual inspection of the trace plot produced by plotting a Euclidean distance at each iteration confirmed convergence of this model parameter.

#### Identifying significant associations

For each analysis, once convergence had been confirmed and the burn-in discarded, posterior probabilities (PPs) were calculated. However, due to the effect of strong linkage disequilibrium (LD), the posterior probability of a QTL may be distributed across several adjacent SNPs. To account for this, and to accurately identify the presence of a QTL, posterior probabilities were also calculated using a sliding window of 5 adjacent SNPs based on genomic location. Subsequent QTL with a PP > 0.5 were defined as high PP QTL. For each trait separately, high PP QTL for each of the eleven analyses (1 - π = 1- pSSR/2, 1 - pSSR, 1 - pSSR × 2 and 0.05-6.25 × 10^−5^) were identified. The number of analyses that a QTL had a PP > 0.5 across the 11 analyses was calculated and assigned to the QTL as its occurrence rate.

For each of the eleven analyses within a trait, an average occurrence rate was calculated by summing the individual QTL occurrence rates of QTL with a PP > 0.5 and dividing this value by the total number of QTL with a PP > 0.5. The analysis with the highest average occurrence rate was then identified (Additional file 5). All QTL with a PP > 0.5 within the analysis with the highest average occurrence rate were then considered significantly associated with the respective trait. This was done for each trait separately, resulting in 4 datasets of significantly associated QTL corresponding to each trait under investigation (Additional file 2). Each dataset represented the analysis with the largest number of frequently occurring high PP QTL for each trait.

### Pathway analysis

Four datasets, corresponding to each trait, were created by identifying all bovine genes within a 500 kb region up and downstream of SNPs located within a QTL significantly associated with a trait using the Bayesian method. To investigate the combined role that some pathways may have on each of these traits, a combined trait dataset containing all genes from each of the individual trait datasets was also created. The genes in each of these five datasets were then mapped to their human orthologs using the mapping available from version hg19 of the human genome. A background set of all possible orthologs that could be represented was created containing all human genes that had a bovine ortholog that was within 500 kb of a SNP included in the analysis (17,186 human genes). For each trait dataset the R [18] package GOseq [19], without the correction for gene length bias, was used to identify the KEGG pathways which were significantly over-represented (p < 0.05) by the set of genes compared against the background set of human genes.

## Results

### Significant associations

#### Carcass weight

**Figure 1:**
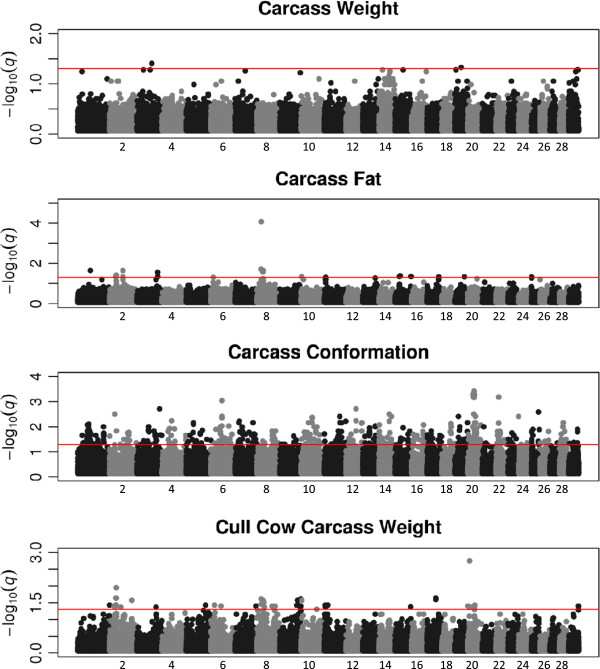
**Genome-wide association results from the single SNP regression are plotted for each trait.** Results for SNPs on all autosomal chromosomes are plotted as negative log transformed q-values. The red continuous line indicates a significance threshold of 1.3 (q < 0.05). Odd numbered chromosomes are plotted in black and even numbered in grey.

Using the SSR method, two QTL were associated (q < 0.05) with carcass weight. These SNPs were on chromosomes 3 and 19 (Figure 1).

In the Bayesian analysis, 11 QTL were associated with carcass weight (Table 2), three of which were located on chromosome 3. Interestingly, two of these QTL, within 2.5 Mb of each other, were located either side of the leptin receptor. Only one of the 11 QTL was associated with both carcass weight and at least one of the other three carcass traits. This QTL, on chromosome 6 (~85 Mb), was associated with both carcass weight and carcass conformation using the Bayesian method. None of the QTL identified as associated with carcass weight were common to both statistical approaches.

**Table 2:** **The number of QTL that were significantly associated with each trait from the single SNP regression (SSR) or Bayesian analysis**

Trait	SSR	Bayesian
Carcass weight	2	11
Carcass fat	24	6
Carcass conformation	414	13
Cull cow carcass weight	46	17

SNPs with a q-value <0.05 from the SSR analysis were considered significantly associated with a trait. Significantly associated adjacent SNPs from the SSR were considered members of the same QTL.

#### Carcass fat

Using the SSR approach, 24 QTL were associated (q < 0.05) with carcass fat (Table 2). The most significantly associated SNP from this analysis (q = 8.45 × 10^−5^), rs109514593, was located within a QTL on chromosome 8 at ~22 Mb (Figure 1), while another SNP (rs41607785), located within a separate QTL, approximately 1 Mb away from rs109514593, was also associated with carcass fat. Five QTL were associated with both carcass fat and cull cow carcass weight. One SNP, rs109776183, was associated with both carcass fat and carcass conformation.

Using the Bayesian method, six QTL were associated with carcass fat. Each of these QTL were located on different chromosomes of the genome. One QTL on chromosome 3 (~105 MB) was associated with carcass fat using both the Bayesian and SSR methods. This SNP was located approximately 600 kb away from rs43359171, which was also associated with carcass fat using the SSR approach.

#### Carcass conformation

A total of 414 QTL were associated (q < 0.05) with carcass conformation in the SSR analysis (Table 2). Significant QTL for carcass conformation were located on all chromosomes (Figure 1). Twenty-one QTL showed a strong association with this trait (q < 0.005), the most significant (q = 3.787 × 10^−4^) of which was on chromosome 20. This SNP, rs41580285, resided within a cluster of 5 strongly associated SNPs (q < 0.005), all of which were less than 1 Mb away from the growth hormone receptor (*GHR*) gene.

Thirteen QTL were associated with carcass conformation in the Bayesian analysis. Seven of these QTL contained at least one SNP that was also associated with carcass conformation using the SSR approach. One of these SNPs was strongly associated with carcass conformation (q < 0.005) using the SSR method. One SNP, located on chromosome 6, was also associated with carcass weight using the Bayesian method.

#### Cull cow carcass weight

A total of 60 QTL were associated with cull cow carcass weight using either the Bayesian or SSR method (Table 2). Of these, 46 QTL were associated (q < 0.05) with cull cow carcass weight using the SSR method (Figure 1). One SNP, rs41935177, was detected as being strongly associated (q < 0.005) with cull cow carcass weight in both the SSR and Bayesian method (PP = 0.95). Seven SNPs from this analysis were associated with both cull cow carcass weight and another carcass trait (5 SNPs were associated with carcass fat and 2 with carcass conformation) using the SSR approach.

Seventeen QTL were associated with cull cow carcass weight in the Bayesian analysis. Three of these QTL, centered on rs109184437, rs41935177 and rs110340777 respectively, were also significantly associated with cull cow carcass weight using the SSR approach.

#### Over-represented KEGG pathways

In total, 557 unique bovine genes were within 500 kb of a QTL associated with a trait using the Bayesian approach (Additional file 6). Of these, 423 mapped to 426 human orthologs. The most significantly over-represented KEGG pathway detected using these genes was the peroxisome proliferator-activated receptor (PPAR) signaling pathway (p = 1.14 × 10^−3^) (Additional file 7). This pathway was significantly over-represented in both carcass fat and the combined trait analyses. In fact, all nine pathways significantly over-represented in the combined trait analysis were also significantly over-represented for a trait when only orthologs from that trait were used in the analysis. Twenty-four different pathways were significantly over-represented across all analyses and are contained in Table 3.

**Table 3:** **Significantly over-represented KEGG pathways and candidate genes**

Trait	Pathway name	p-value	Candidate genes
CWT	Jak-STAT signaling pathway	0.00503	*IL12RB2*, *IL23R*, *JAK1*, *LEPR*
CWT	Cell cycle	0.01768	*ANAPC1*, *GADD45A*, *SMC1B*
CWT	Metabolism of xenobiotics by cytochrome P450	0.01861	*ALDH1A3*, *CYP1B1*
CWT	p53 signaling pathway	0.03380	*GADD45A*, *GTSE1*
CWT	Adipocytokine signaling pathway	0.04000	*LEPR*, *PPARA*
CWT	Sulfur relay system	0.04560	*TRMU*
CFAT	PPAR signaling pathway	0.00114	*CYP4A11*, *CYP4A22*, *FADS2*
CFAT	Protein digestion and absorption	0.00129	*PGA3*, *PGA4*
CFAT	Biosynthesis of unsaturated fatty acids	0.00165	*FADS1*, *FADS2*
CFAT	Vascular smooth muscle contraction	0.00460	*CYP4A11*, *CYP4A22*
CFAT	Fatty acid metabolism	0.00716	*CYP4A11*, *CYP4A22*
CFAT	Retinol metabolism	0.00854	*CYP4A11*, *CYP4A22*
CFAT	Glycerolipid metabolism	0.00890	*AGPAT4*, *DAK*
CFAT	Arachidonic acid metabolism	0.01081	*CYP4A11*, *CYP4A22*
CFAT	Non-homologous end-joining	0.03864	*FEN1*
CONF	Inositol phosphate metabolism	0.01392	*INPP5B*, *PI4KB*, *PIP5K1A*
CONF	Biotin metabolism	0.01727	*HLCS*
CONF	Lysosome	0.01892	*CTSK*, *CTSS*, *LAMP3*, *MAN2B1*
CONF	Phosphatidylinositol signaling system	0.03030	*INPP5B*, *PI4KB*, *PIP5K1A*
CONF	Intestinal immune network for IgA production	0.04489	*TNFSF13B*
CONF	Proteasome	0.04913	*PSMB4*, *PSMD4*
CULL	Mucin type O-Glycan biosynthesis	0.00178	*GALNT8*, *GALNTL2*, *GALNTL6*
CULL	Vibrio cholerae infection	0.00981	*ATP6V1C2*, *GNAS*, *KCNQ1*
CULL	p53 signaling pathway	0.01664	*CCNB1*, *CCND2*, *RRM2*
CULL	Gap junction	0.03030	*GNAS*, *MAP3K2*, *TUBB1*
ALL	PPAR signaling pathway	0.00672	*SLC27A6*, *FADS2*, *CYP4A22*, *RXRA*, *PPARA*, *CYP4A11*
ALL	Phosphatidylinositol signaling system	0.01284	*DGKD*, *PI4KB*, *PIP5K1A*, *INPP5D*, *INPP5B, CALM1*
ALL	p53 signaling pathway	0.01862	*GTSE1*, *GADD45A*, *CCND2*, *CCNB1*, *RRM2*
ALL	Mucin type O-Glycan biosynthesis	0.03132	*GALNT8*, *GALNTL2*, *GALNTL6*
ALL	Protein digestion and absorption	0.03135	*KCNQ1*, *KCNJ13*, *PGA4*, *PGA3*
ALL	Vibrio cholerae infection	0.03729	*KCNQ1*, *SLC12A2*, *GNAS*, *ATP6V1C2*
ALL	Arachidonic acid metabolism	0.03964	*PTGDS*, *CBR3*, *CYP4A22*, *CYP4A11*
ALL	Non-homologous end-joining	0.03992	*FEN1*, *LIG4*
ALL	Biotin metabolism	0.04896	*HLCS*

Candidate genes are genes that occurred in the over-represented pathway and were within 500kbs of a QTL significantly associated with the trait using the Bayesian approach.

CWT = carcass weight; CFAT = carcass fat; CONF = carcass conformation; CULL = cull cow carcass weight; ALL = significantly over-represented KEGG pathways using combined trait gene dataset.

## Discussion

The aim of the study was to identify regions of the bovine genome associated with carcass characteristics using phenotypes of four economically important carcass traits in Holstein-Friesian cattle: carcass weight, carcass fat, carcass conformation of progeny as well as cull cow carcass weight. Two statistical approaches, a Bayesian and frequentist, were used to detect associations between SNPs and each of the traits separately. Detected SNP associations using either approach were distributed across all autosomes.

### The Bayesian approach

Both the Bayesian and SSR methods differ fundamentally in their underlying approaches. The single SNP regression method tests each SNP individually, whereas the Bayesian approach tests one SNP at a time while taking cognizance of all other SNPs simultaneously. This was particularly evident by the Bayesian approach identifying a single marker whereas the SSR approach sometimes identified a cluster of adjacent significant associations for the same location (e.g. chromosome 20 at ~10 MB for cull cow carcass weight); this a consequence of linkage disequilibrium in the genome. Also, the Bayesian approach is advantageous as there is no need to correct for Type I errors arising from many thousands of tests. This allowed us to detect associations that might have been removed as false positives by the multiple testing correction method applied to the SSR approach. In fact, 40 of 47 QTL identified from the Bayesian approach were also significantly associated (p < 0.05) with the same trait using the SSR before correction for multiple tests. After correcting for multiple testing, this number dropped to 11. Furthermore, as complex traits are likely to be influenced by a large number of mutations, models that analyze all markers simultaneously should provide more accurate results than models that analyze one or a few markers independently [20]. Thus Bayesian approaches may then have greater power to detect SNPs with moderate effects on a trait of interest. Additionally, the ability to incorporate information *a priori* into the model would appear to be advantageous in complex traits that are influenced by many variants. Although inclusion of a prior may bias results to fit that prior [21], it is likely that SNPs with the strongest association will be identified irrespective of the prior proportion of SNPs assumed to have an effect. However, this cannot be guaranteed and as such, should be investigated as is the case in this study. Our choice of prior would appear to be robust, as it represents the most frequently occurring high PP QTL across different prior specifications.

In MCMC Bayesian approaches it is necessary to ensure that the chains have converged before calculating posterior probabilities [22–24]. This can be done in several ways. For instance previous GWAS analyses using Bayesian approaches have used the convergence of the SNP effect for selected SNPs as evidence of model convergence [25]. This however is only a single parameter and its convergence may not represent the convergence of the entire model. As discussed by Cowles and Carlin (1996) [23], there is no one conclusive diagnostic that can provide assurance of convergence. Convergence of all parameters, not just those of interest, should be checked before making any posterior inferences [26]. With some models, certain parameters can appear to exhibit good convergence behavior. This however, can be misleading due to the slow convergence of other parameters [26]. To tackle this problem, we used the convergence of the sum of log-likelihoods for all SNPs at each iteration and identified when this converged. In addition to this, the total number of high PP (PP > 0.5) SNPs and the Euclidean distance between SNP effects estimated from the Bayesian and SSR approaches for these SNPs were plotted at every sampled iteration. This was to ensure that the MCMC chains had run long enough and that the model had successfully converged.

### Significant associations

A large number of associations (514 QTL) were detected across all traits using both statistical approaches. However, most of these were detected for carcass conformation (414) using the SSR approach (q < 0.05). This may be due to biological noise caused by an increased complexity of this trait compared to the others analyzed, or because the trait may be more greatly influenced by several other unmeasured physical characteristics such as bone size and carcass frame. At a significance of q < 0.005, a total of 21 QTL were associated with carcass conformation using the SSR approach. This figure was much more similar to the results from the other three traits. Using this significance threshold for carcass conformation and a significance threshold of q < 0.05 for the other three traits, 90 QTL were associated with at least one trait using the SSR approach. This meant that 129 QTL were associated with at least one of the traits using both the SSR and Bayesian approaches.

### Candidate genes

Using both statistical approaches, a number of associations detected for each trait were in close proximity to genes with a known role in animal growth (e.g. growth hormone receptor (*GHR*), Insulin and Insulin-like growth factor 2 (*IGF2*)). As well as this, a number of novel candidate genes were identified. For example, significant QTL on chromosome 20 were detected within 1 MB of fibroblast growth factor 11 (*FGF11*) and on chromosome 6 approximately 500 kb away from Gonadotropin-releasing hormone receptor.

### Glucagon gene

Three novel associations with carcass fat were detected on chromosome 2, all of which were within a 3.5 Mb region upstream of the glucagon gene. In the same region, 5 SNPs that were associated with cull cow carcass weight were all within a 2.9 Mb region of the glucagon gene. The glucagon gene plays an important role in a number of biological processes related to metabolism and energy homeostasis [27]. Glucagon is known to regulate fat metabolism via cAMP-dependent mechanisms in animals [27].

### Leptin gene

A number of associations detected from the Bayesian approach, that were not detected in the SSR approach, occurred in regions containing genes previously reported to be associated to growth in Holstein cows (e.g. leptin gene [28]). Interestingly, associations from the Bayesian method that were not detected using the SSR approach, also occurred in close proximity to the leptin receptor (approx. 300 kb upstream). A mutation in the leptin receptor has previously been reported to cause obesity in humans [29]. Leptin is involved in the hypothalamic control of energy homeostasis, an indicator of body fat reserves and regulator of energy expenditure [30]. In ruminants, such as cattle, a positive correlation has been demonstrated between circulating concentrations of leptin and fat accumulation [31].

### Over-represented KEGG pathways

Carcass traits are governed by many complex biological systems, reflecting the combined influence of many genetic factors. However, there may be central biological processes that link together the genetic regulation of all of these traits. The combined trait analysis detected biological pathways that were not found using the individual trait datasets (e.g. peroxisome proliferator-activated receptor signaling pathway). These biological pathways contained genes associated with several different carcass traits, and are thus likely involved in different aspects of each of these traits.

### Peroxisome proliferator-activated receptor

**Figure 2:**
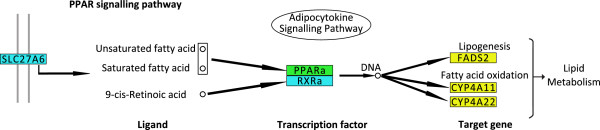
**Genes from the PPAR signaling pathway that were in regions surrounding QTL associated with at least one trait using the Bayesian approach.** Genes that are colored in blue, green and yellow were within 500kbs of a QTL associated with carcass conformation, carcass weight and carcass fat, respectively. The complete figure of the PPAR signaling pathway, showing all genes in this pathway, is contained in Additional file 7.

Peroxisome proliferator-activated receptor (PPAR) signaling pathway was the most significantly over-represented pathway (p = 0.00114) in both the analysis involving carcass fat and the combined trait dataset (p = 0.00672). PPARs are a group of transcription factors that play an essential physiological role in the regulation of adipocyte tissue development, lipogenesis and skeletal muscle lipid metabolism [32–34]. There are three members of the PPAR family, *PPARα*, *PPARγ* and *PPARδ*, each of which is encoded by a separate gene [35]. PPARs regulate transcription by binding with retinoid X receptors [36]. This heterodimer binds to peroxisome proliferator response elements in the promoter region of target genes, which then stimulates expression [37]. Both *PPARα* and retinoid X receptor α were identified as candidate genes involved in regulating carcass weight and carcass conformation, respectively. Furthermore, *PPARα* is also involved in controlling the expression of fatty acid binding proteins, which are a family of carrier proteins involved in mediating intracellular uptake and transport of long-chain fatty acids within the cell [38, 39]. Fatty acid binding proteins also play an important role in systemic energy homeostasis [40]. Interestingly, genes from the carcass weight, carcass fat and carcass conformation gene datasets were also in this pathway suggesting that PPAR may also play a role in each of these traits (Figure 2). This was not unexpected given the known genetic associations among these traits [41].

### Phosphatidylinositol signaling system

Phosphoinositides are a family of minor membrane lipids involved in signal transduction, which play important roles in several signaling pathways within the cell [42]. Phosphoinositides initiate signaling by specifically interacting with a large number of proteins that can result in relocalization of the protein from one area of the cell to another, or induce conformational changes in the protein [42, 43]. The immediate precursor to all phosphoinositides is phosphatidylinositol [44, 45]. Signaling through various phosphoinositides has also been implicated in a wide range of cellular processes including cell growth and proliferation, apoptosis and intracellular vesicle trafficking [42, 43, 46, 47]. The phosphatidylinositol signaling system is initiated in response to environmental stimuli such stress and diet. This pathway was significantly over-represented for both the carcass conformation (p = 0.0303) and the combined trait datasets (p = 0.01284) (Additional file 8). Interestingly, candidate genes from the carcass weight, carcass fat and carcass conformation gene datasets were also in this pathway. This is not surprising given the wide range of functions that phosphatidylinositol signaling has been implicated in. Furthermore, genes involved in the phosphatidylinositol signaling system have been found to differentially expressed in studies examining growth and fatness traits in pigs [34]. This pathway, along with pathways significantly over-represented from the combined trait dataset, may contain core biological processes linked to phenotypic variation observed in each of the traits under investigation.

### Conserved biological functions

There are numerous examples of single genes (or mutations in a gene) influencing similar phenotypes in different species. Some well known examples include mutations in the myostatin gene that lead to the “double muscling” phenotype in humans [48], mice [49] and cattle [8, 9]. Another example is the control of hair color by the melanocortin receptor gene (*Mc1r*) in humans [50], with similar effects on coat color in species such as cattle [51], pigs [52] and horses [53]. For complex traits, there is little known on the conservation of genes with low to moderate effects on a phenotype across species. However, there are a number of examples that suggest a degree of conservation of gene classes between mammalian species (*e.g.* stature [54] and milk proteins [55]) exists [54]. From our study, we have identified some well-known biological processes that influence similar traits in humans such as PPAR signaling and its influence in fat deposition and metabolism [56]. In fact, several of the pathways identified in our study have reported roles in similar traits in other organisms. For example, arachidonic acid metabolism has been linked to increased adipose tissue development in infant mice [57]. In addition, levels of arachidonic acid content in adipose tissue have been shown to be higher in overweight and obese children [58]. It is not surprising then, that this pathway was significantly over-represented for carcass fat. A number of pathways with a novel association in cattle, but with known effects in other organisms have also been identified (e.g. Jak-STAT signaling pathway). The Jak-STAT signaling pathway plays an important role in several processes related to cell proliferation, differentiation, migration and apoptosis [59]. This pathway is also highly conserved across species [60], and has been linked to skeletal muscle development in mice [61] and humans [62]. This would suggest that a number of the biological processes influencing growth characteristics that are conserved in organisms such as humans are also conserved in cattle.

## Conclusions

In the present study, a large number of significant associations, candidate regions, and biological pathways were identified using two different statistical approaches. The use of a Bayesian approach facilitated the identification of associations that might have been removed from the SSR analysis as a false positive after correcting for multiple testing.

Bayesian approaches would seem to have merit in future association studies as they provide numerous advantages over linear regression approaches such as avoiding many thousands of tests by fitting all of the data at once and allowing the inclusion of information *a priori*. However, including information *a priori* may create bias that influences posterior inferences. As such, exploring a dispersion of prior specifications and combining this information may reduce bias towards to a single arbitrarily chosen prior [63]. Furthermore, correctly identifying convergence of a Bayesian approach will remain a contentious subject. Monitoring the behavior of numerous model parameters, not just those of interest, as is the case in this study, will provide the best opportunity to confidently confirm convergence when using a Bayesian approach.

A large number of significant associations were detected in this analysis. These associations can help to further refine known large QTL regions and support the identification of any underlying causative mutations. Also, the gene datasets created within this study may form the basis of further investigation, utilizing next-generation sequencing technologies, for targeted re-sequencing which may yield a panel of potential causative mutations. Furthermore, a number of biological pathways with a known role in organisms such as humans and mice were identified as having a function in similar analogous traits in *Bos taurus*. This supports previous findings which suggest that several core biological processes involved in growth and metabolism are highly conserved across species. In particular, the PPAR signaling pathway would appear to have a key role in controlling several aspects of bovine growth. However, further investigation to understand the cumulative influence that gene interactions have and the multi-faceted role that PPAR and other core biological pathways have on phenotypic expression of growth and carcass traits is warranted.

## Electronic supplementary material

Additional file 1: **Animal Identifiers for all animals used in this study.** These identifiers can be used to request information from the Irish Cattle Breeding Federation database (http://www.icbf.com). (XLS 70 KB)

Additional file 2: **SNP information for all SNPs included in the analysis.** This file contains the chromosome number, position and dbSNP identifier for all SNPs included in either the single SNP regression or Bayesian analyses. The q-values for all SNPs included in the single SNP regression analysis for each trait are also included. Posterior probabilities, estimated using a 5 SNP sliding window, for the Bayesian analysis are also included in adjacent columns. (CSV 4 MB)

Additional file 3: **The maximum number of iterations that each Bayesian analysis was run for.** (DOC 45 KB)

Additional file 4: **Initial iterations discarded as burn-in from each Bayesian analysis.** (DOC 44 KB)

Additional file 5: **The average occurrence rate of high PP QTL for each Bayesian analysis.** (DOC 46 KB)

Additional file 6: **Ensembl gene IDs for all Bovine genes within 500 kbs of a QTL associated with a trait using the Bayesian approach.** This file also contains the human ortholog(s) for each bovine gene and which of the trait datasets it was assigned to. (CSV 21 KB)

Additional file 7: **The peroxisome proliferator-activated receptor (PPAR) signaling pathway.** PPAR was the most significantly over-represented KEGG pathway in the combined trait analysis. Genes in this pathway were in regions surrounding QTL associated to three different traits using the Bayesian approach (colored in red). (PDF 558 KB)

Additional file 8: **The phosphatidylinositol signaling system.** This pathway was significantly over-represented in the carcass conformation and combined trait analyses. Genes from this pathway that were within 500 kbs of significantly associated QTL using the Bayesian approach are highlighted in red. (PDF 534 KB)
