# Supplementary material for: Whole genome association study identifies regions of the bovine genome and biological pathways involved in carcass trait performance in Holstein-Friesian cattle
Source: BMC Genomics. 2014 Oct 1;15(1):837. doi: 10.1186/1471-2164-15-837 (PMC4192274; doi:10.1186/1471-2164-15-837)
Supplement: Supplementary file 3 — Additional file 3: The maximum number of iterations that each Bayesian analysis was run for. (DOC 45 KB) [file 12864_2013_6513_MOESM3_ESM.doc]

**Additional file 3** **Maximum iterations each Bayesian analysis was run for.**

| **1 - π** | **CWT** | **CFAT** | **CONF** | **CULL** |
| --- | --- | --- | --- | --- |
| 1 - pSSR/2 | 800,000 | 950,000 | 850,000 | 700,000 |
| 1 - pSSR | 800,000 | 950,000 | 850,000 | 700,000 |
| 1 - pSSR*2 | 800,000 | 950,000 | 850,000 | 700,000 |
| 6.25×10^-5^ | 600,000 | 700,000 | 600,000 | 700,000 |
| 1.25×10^-4^ | 600,000 | 700,000 | 600,000 | 700,000 |
| 2.5×10^-4^ | 550,000 | 650,000 | 600,000 | 700,000 |
| 5.0×10^-4^ | 600,000 | 700,000 | 600,000 | 700,000 |
| 1.0×10^-3^ | 400,000 | 500,000 | 400,000 | 450,000 |
| 2.45×10^-3^ | 450,000 | 500,000 | 400,000 | 500,000 |
| 1.0×10^-2^ | 400,000 | 500,000 | 400,000 | 550,000 |
| 5.0×10^-2^ | 400,000 | 500,000 | 400,000 | 500,000 |

(1 – π) = prior proportion of SNPs assumed to be associated with a trait; pSSR = the proportion of SNPs not significant from single SNP regression analysis. One minus this value is the prior proportion of SNPs assumed to be associated with each trait; CWT = carcass weight; CFAT = carcass fat; CONF = carcass conformation; CULL = cull cow carcass weight
