# Supplementary material for: Whole genome association study identifies regions of the bovine genome and biological pathways involved in carcass trait performance in Holstein-Friesian cattle
Source: BMC Genomics. 2014 Oct 1;15(1):837. doi: 10.1186/1471-2164-15-837 (PMC4192274; doi:10.1186/1471-2164-15-837)
Supplement: Supplementary file 4 — Additional file 4: Initial iterations discarded as burn-in from each Bayesian analysis. (DOC 44 KB) [file 12864_2013_6513_MOESM4_ESM.doc]

**Additional file 4** **Initial iterations discarded as burn-in from each Bayesian analysis.**

| **1 - π** | **CWT** | **CFAT** | **CONF** | **CULL** |
| --- | --- | --- | --- | --- |
| 1 - pSSR/2 | 107,000 | 126,500 | 102,500 | 192,000 |
| 1 - pSSR | 122,000 | 102,000 | 163,500 | 109,500 |
| 1 - pSSR*2 | 277,000 | 77,000 | 211,500 | 54,000 |
| 6.25×10^-5^ | 132,000 | 63,500 | 58,000 | 65,000 |
| 1.25×10^-4^ | 135,500 | 74,500 | 58,500 | 136,500 |
| 2.5×10^-4^ | 213,000 | 99,500 | 102,000 | 182,500 |
| 5.0×10^-4^ | 175,000 | 128,500 | 60,000 | 83,500 |
| 1.0×10^-3^ | 52,500 | 130,500 | 90,000 | 131,500 |
| 2.45×10^-3^ | 57,500 | 94,500 | 120,500 | 69,500 |
| 1.0×10^-2^ | 106,000 | 121,000 | 73,000 | 50,500 |
| 5.0×10^-2^ | 51,500 | 150,500 | 195,000 | 156,000 |

(1 – π) = prior proportion of SNPs assumed to be associated with a trait; pSSR = the proportion of SNPs not significant from single SNP regression analysis. One minus this value is the prior proportion of SNPs assumed to be associated with each trait; CWT = carcass weight; CFAT = carcass fat; CONF = carcass conformation; CULL = cull cow carcass weight
