# Supplementary material for: Whole genome association study identifies regions of the bovine genome and biological pathways involved in carcass trait performance in Holstein-Friesian cattle
Source: BMC Genomics. 2014 Oct 1;15(1):837. doi: 10.1186/1471-2164-15-837 (PMC4192274; doi:10.1186/1471-2164-15-837)
Supplement: Supplementary file 5 — Additional file 5: The average occurrence rate of high PP QTL for each Bayesian analysis. (DOC 46 KB) [file 12864_2013_6513_MOESM5_ESM.doc]

**Additional file 5** **Average occurrence rate of high PP SNPs for each Bayesian analysis.**

| **1 - π** | **CWT** | **CFAT** | **CONF** | **CULL** |
| --- | --- | --- | --- | --- |
| 1 - pSSR/2 | 4.71 | 2.54 | 1.66 | 4.28 |
| 1 - pSSR | 6.00 | 2.11 | 1.60 | 5.24 |
| 1 - pSSR*2 | 6.14 | 2.11 | 1.43 | 3.83 |
| 6.25×10^-5^ | 6.91 | 3.50 | 3.92 | 2.69 |
| 1.25×10^-4^ | 4.50 | 2.00 | 3.10 | 3.85 |
| 2.5×10^-4^ | 5.10 | 2.85 | 2.55 | 4.25 |
| 5.0×10^-4^ | 3.30 | 2.30 | 2.47 | 4.61 |
| 1.0×10^-3^ | 3.79 | 1.69 | 2.32 | 3.25 |
| 2.45×10^-3^ | 3.00 | 1.67 | 1.81 | 3.21 |
| 1.0×10^-2^ | 2.43 | 1.73 | 1.45 | 3.48 |
| 5.0×10^-2^ | 1.34 | 1.03 | 1.14 | 1.14 |

(1 – π) = prior proportion of SNPs assumed to be associated with a trait; pSSR = the proportion of SNPs not significant from single SNP regression analysis. One minus this value is the prior proportion of SNPs assumed to be associated with each trait; CWT = carcass weight; CFAT = carcass fat; CONF = carcass conformation; CULL = cull cow carcass weight
