# Supplementary figures and images for: Whole genome association study identifies regions of the bovine genome and biological pathways involved in carcass trait performance in Holstein-Friesian cattle
Source: BMC Genomics. 2014 Oct 1;15(1):837. doi: 10.1186/1471-2164-15-837 (PMC4192274; doi:10.1186/1471-2164-15-837)

# PPAR SIGNALING PATHWAY

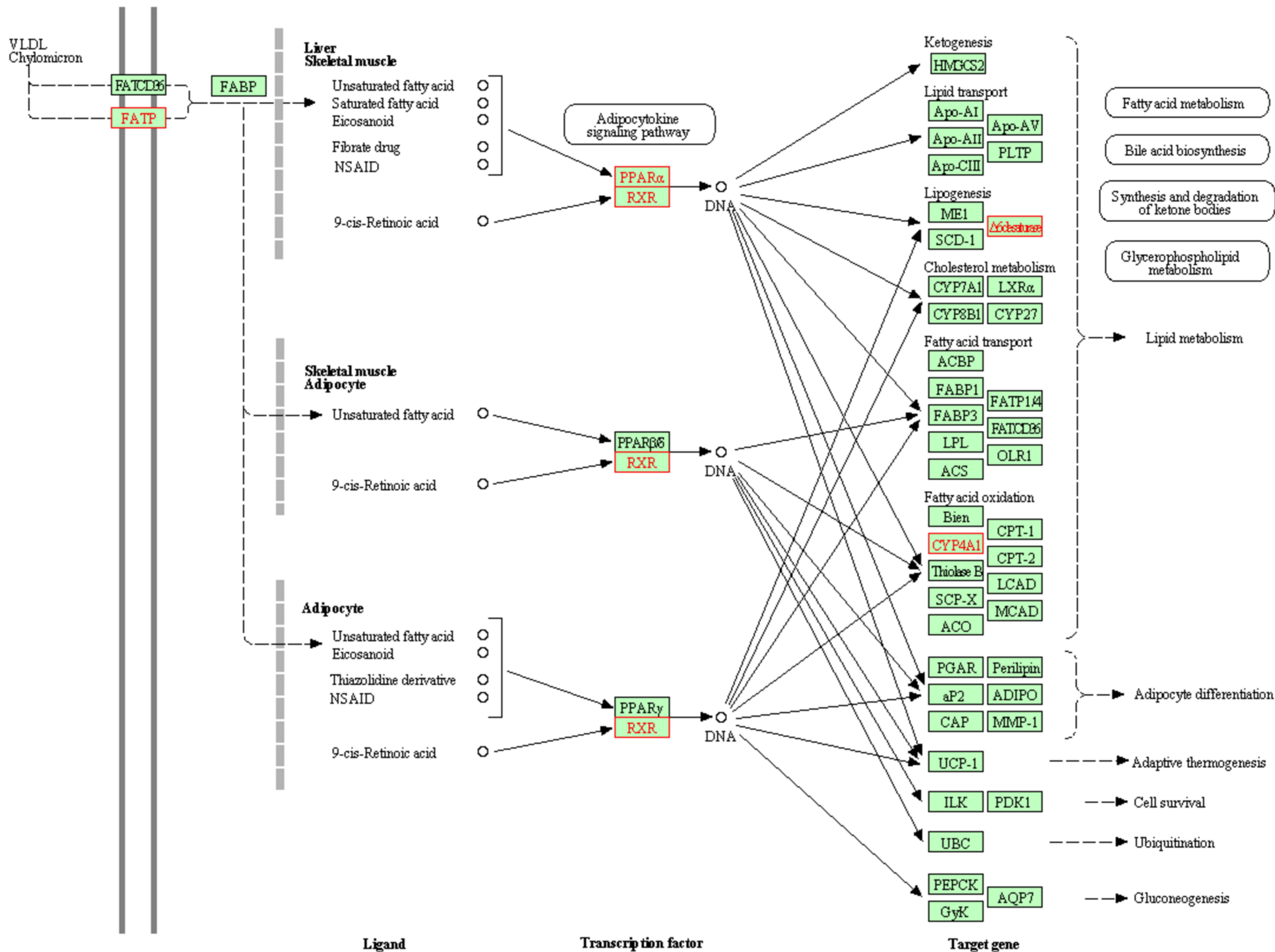

Supplement: Supplementary file 7 — Additional file 7: The peroxisome proliferator-activated receptor (PPAR) signaling pathway. PPAR was the most significantly over-represented KEGG pathway in the combined trait analysis. Genes in this pathway were in regions surrounding QTL associated to three different traits using the Bayesian approach (colored in red). (PDF 558 KB) [file 12864_2013_6513_MOESM7_ESM.pdf]

## PHOSPHATIDYLINOSITOL SIGNALING SYSTEM

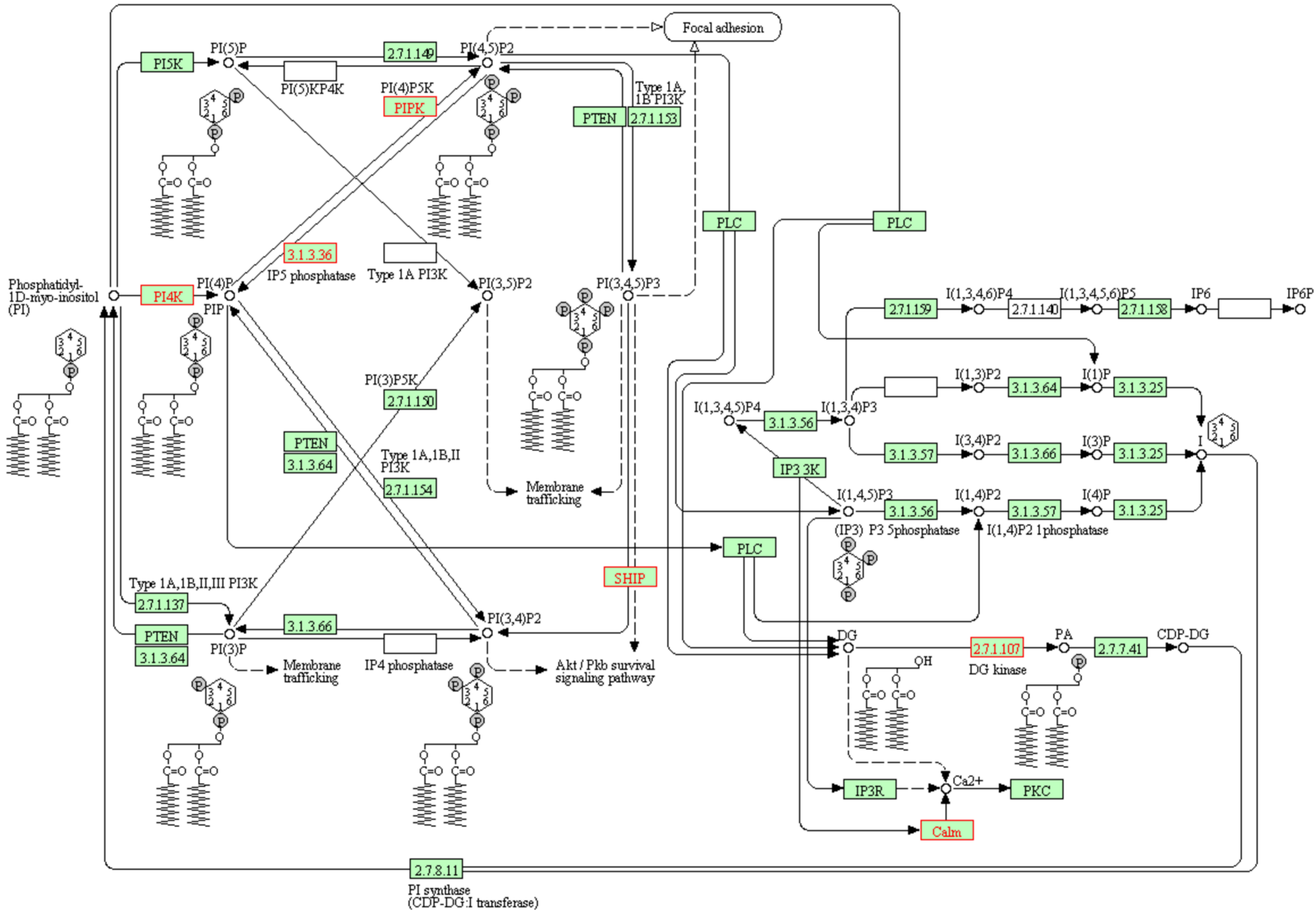

Supplement: Supplementary file 8 — Additional file 8: The phosphatidylinositol signaling system. This pathway was significantly over-represented in the carcass conformation and combined trait analyses. Genes from this pathway that were within 500 kbs of significantly associated QTL using the Bayesian approach are highlighted in red. (PDF 534 KB) [file 12864_2013_6513_MOESM8_ESM.pdf]
